# Freshwater protected areas can preserve high-performance phenotypes in populations of a popular sportfish

**DOI:** 10.1093/conphys/coad004

**Published:** 2023-03-16

**Authors:** A J Zolderdo, A E I Abrams, M J Lawrence, C H Reid, C D Suski, K M Gilmour, S J Cooke

**Affiliations:** Fish Ecology and Conservation Physiology Laboratory, Department of Biology and Institute of Environmental and Interdisciplinary Science, Carleton University, Ottawa, ON, Canada; Queen’s University Biological Station, Queen’s University, Kingston, ON, Canada; Fish Ecology and Conservation Physiology Laboratory, Department of Biology and Institute of Environmental and Interdisciplinary Science, Carleton University, Ottawa, ON, Canada; Fish Ecology and Conservation Physiology Laboratory, Department of Biology and Institute of Environmental and Interdisciplinary Science, Carleton University, Ottawa, ON, Canada; Fish Ecology and Conservation Physiology Laboratory, Department of Biology and Institute of Environmental and Interdisciplinary Science, Carleton University, Ottawa, ON, Canada; Department of Natural Resources and Environmental Sciences, University of Illinois Urbana-Champaign, Urbana, IL, USA; Department of Biology, University of Ottawa, 30 Marie Curie, Ottawa, ON, Canada; Fish Ecology and Conservation Physiology Laboratory, Department of Biology and Institute of Environmental and Interdisciplinary Science, Carleton University, Ottawa, ON, Canada

**Keywords:** recreational angling, metabolism, largemouth bass, fisheries-induced evolution, Cortisol

## Abstract

Recreational fishing has the potential to cause evolutionary change in fish populations; a phenomenon referred to as fisheries-induced evolution. However, detecting and quantifying the magnitude of recreational fisheries selection in the wild is inherently difficult, largely owing to the challenges associated with variation in environmental factors and, in most cases, the absence of pre-selection or baseline data against which comparisons can be made. However, exploration of recreational fisheries selection in wild populations may be possible in systems where fisheries exclusion zones exist. Lakes that possess intra-lake freshwater protected areas (FPAs) can provide investigative opportunities to evaluate the evolutionary impact(s) of differing fisheries management strategies within the same waterbody. To address this possibility, we evaluated how two physiological characteristics (metabolic phenotype and stress responsiveness) as well as a proxy for angling vulnerability, catch-per-unit-effort (CPUE), differed between populations of largemouth bass (*Micropterus salmoides*) inhabiting long-standing (>70 years active) intra-lake FPAs and adjacent, open access, main-lake areas. Fish from FPA populations had significantly higher aerobic scope (AS) capacity (13%) and CPUE rates compared with fish inhabiting the adjacent main-lake areas. These findings are consistent with theory and empirical evidence linking exploitation with reduced metabolic performance, supporting the hypothesis that recreational fishing may be altering the metabolic phenotype of wild fish populations. Reductions in AS are concerning because they suggest a reduced scope for carrying out essential life-history activities, which may result in fitness level implications. Furthermore, these results highlight the potential for unexploited FPA populations to serve as benchmarks to further investigate the evolutionary consequences of recreational fishing on wild fish and to preserve high-performance phenotypes.

## Teaser

The findings presented here showcase how freshwater protected areas, where all forms of fisheries are prohibited, can provide physiological benefits and improved catchability in wild populations of largemouth bass. These findings support the use of freshwater protected area as an effective conservation strategy to improve contemporary fisheries management and conservation practices.

## Introduction

Recreational fishing is an important sport and leisure activity practiced globally in both freshwater and marine environments, and accounts for an estimated 12% of annual global fish harvest (Cooke and Cowx, 2004). Recently, however, there has been concern regarding potential evolutionary consequences of recreational fishing on wild fish populations. Similar to commercial fishing, recreational fishing has the selective potential to alter the phenotypes of highly exploited populations, a phenomena referred to as fisheries-induced evolution (FIE; Heino and Dieckmann, 2009). FIE occurs due to intense selection pressure on specific phenotypes within a population (Kuparinen and Merilä, 2007; Heino and Dieckmann, 2008). Selection in this context arises through harvesting with fishing gear and tactics that target fish of a particular size class, sex, life-history stage, and/or behaviour (reviewed in Diaz Pauli and Sih, 2017; Wang *et al.*, 2016). As such, selection imparted through fisheries can induce evolutionary changes that oppose natural selection processes (Miller, 1957; Sih *et al.*, 2004; Nussle *et al.*, 2016; Hollins *et al.*, 2018). Furthermore, the altered fish population(s) may be less desirable for recreational angling owing to increased timidity which impacts catch-per-unit-effort (CPUE) (Alós *et al.*, 2012; Philipp *et al.*, 2015).

Two key physiological traits that may be altered by fisheries practices are metabolism and the responsiveness of the stress axis (i.e. stress responsiveness). Specifically, fish with greater metabolic demands have a higher propensity to forage/feed owing to increased nutritional requirements, which may increase their likelihood of interacting with fishing lures (Redpath *et al.*, 2010; Hessenauer *et al.*, 2015; Killen *et al.*, 2015). Furthermore, fish that are less sensitive to external stimuli and/or stressors (e.g. fishing gear) may indirectly increase their exposure to angling capture through a reduced fear or caution toward fishing lures (Louison *et al.*, 2017; Hollins *et al.*, 2018).The pace-of-life syndrome (POLS) links both metabolic output and stress responsiveness (i.e. hypothalamic–pituitary-interrenal-axis [HPI] reactivity) to a suite of highly correlated life-history traits (e.g. growth rates, age at maturity, reproductive investment; Réale *et al.*, 2010). These traits are largely influenced through correlational selection, whereby selection pressure on a specific trait has the potential to indirectly alter interconnected traits along the fast-slow pace of life spectrum (Réale *et al.*, 2010; Polverino *et al.*, 2018; Wright *et al.*, 2019). As a result, selective angling practises on a particular life-history trait(s) can lead to phenotypic changes at the population level (Heino and Dieckmann, 2008; Diaz Pauli and Sih, 2017; Hollins *et al.*, 2018). For example, Philipp *et al.* (2009) showed that vulnerability to angling capture (measured via CPUE) is indeed a heritable trait (*h^2^* = 0.146) in largemouth bass (*Micropterus salmoides*), and that vulnerability to angling is correlated with a suite of physiological and behavioural phenotypes (e.g. increased metabolism and parental aggression; reviewed in Philipp *et al.*, 2015). Consequently, selection pressure resulting in changes to HPI reactivity and/or metabolism may also indirectly select for phenotypic changes in life-history traits (e.g. reproductive investment) through pace-of-life mechanisms, resulting in fitness level impacts at the population level (Réale *et al.*, 2010).

To date, research into the selective potential of recreational angling has been largely laboratory based, evaluating captive or hatchery-bred animals under highly controlled experimental conditions (Hessenauer *et al.*, 2015; Philipp *et al.*, 2015; Louison *et al.*, 2017). Although these experimental studies have advanced the mechanistic understanding of the selective potential associated with hook-and-line angling practises, how this selective potential translates to wild populations, under natural condition, is not well understood. Evaluating fisheries-induced selection in wild fish populations is challenging given the various abiotic (e.g. habitat loss) and biotic (e.g. density-dependent resource balancing) factors that may influence the magnitude and extent of a selective force (Stokes *et al.*, 1993; Law, 2000). It is also important to note that pre-selection or baseline data are lacking for most systems, making it difficult to detect or accurately measure the true extent of a particular selective force over time (Law, 2000; Kuparinen and Merilä, 2007). Basing selection inferences on populations originating from different lake systems and exposed to differing fisheries practises can provide unreliable results given the potential differences in environmental factors between lakes (Stokes *et al.*, 1993). Collectively, all of these factors constrain our ability to define the selective impact of fisheries practises in the wild (Reznick *et al.*, 1990; Kuparinen and Merilä, 2007; Stepien *et al.*, 2017).

Despite these challenges, exploration of fisheries-based selection in wild populations may be possible in systems where fisheries exclusion zones (e.g. freshwater protected areas; FPAs) exist. Lakes that possess intra-lake FPAs may provide investigative opportunities to evaluate the impact of differing fisheries management strategies (e.g. FPAs vs open exploitation areas) within the same waterbody, thus providing a whole-lake experimental arena governed by similar ecosystem processes (Suski and Cooke, 2007; Dunlop *et al.*, 2009; Twardek *et al.*, 2017). FPAs that exclude fisheries practises may provide a natural refuge against fisheries-induced selection pressures, enabling a proportion of a targeted population to re-establish a natural state (Bergseth *et al.*, 2016), while the remainder of the population, inhabiting the non-protected lake area(s), may still be subjected to fisheries pressure, potentially creating a directional shift in targeted phenotypes over time (Diaz Pauli and Sih, 2017; Hollins *et al.*, 2018). In short, lakes with FPAs may provide a study system to investigate the selective potential of recreational fisheries in the wild while controlling for ecosystem factors.

**Figure 1:**
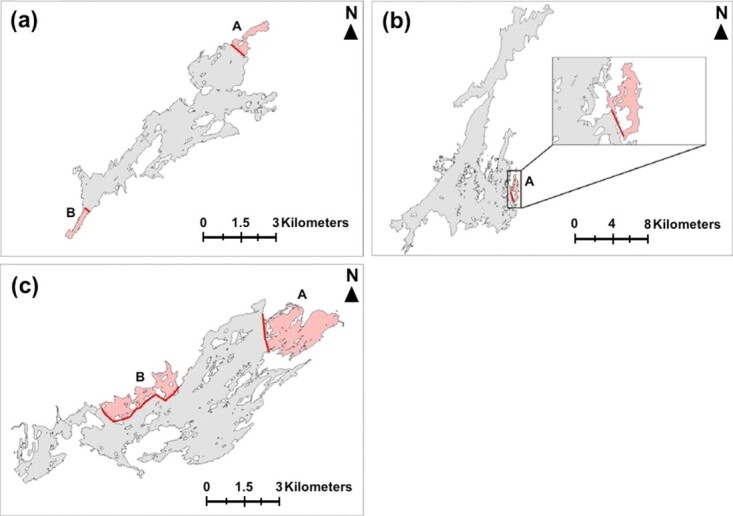
Maps of the three replicate study lakes including OP (a), BR lake (b), and NB Lake (c), adapted from Zolderdo *et al.*, 2019. The freshwater protected areas (FPAs) are designated by red shading, with a solid red line delineating the FPAs borders. For OP and NB lake that have two designated FPAs each (distinguished by ‘A’ and ‘B’, respectively), the data collected from each FPA were pooled together to provide a holistic representation of the protective potential provided by FPAs within each lake system.

There were two objectives for the current study. The first objective was to determine whether the physiological traits of wild largemouth bass differed inside versus outside the FPAs. The second objective was to determine whether the presence of an FPA influenced CPUE (capture rate) of largemouth bass residing inside versus outside the FPAs. Using a series of lakes that contains long-established intra-lake FPAs (>70 years), we addressed the first objective by evaluating the metabolic phenotype, HPI axis-reactivity (cortisol responsiveness), and glucose responsiveness of largemouth bass residing inside versus outside three different FPAs. Vulnerability to angling has been linked to a suite of correlated physiological traits associated with fast POLS characteristics, including high activity phenotypes (i.e. high metabolic performance and low HPI axis-reactivity; Philipp *et al.*, 2009; Alós *et al.*, 2012; Louison *et al.*, 2017). Based on the experimental literature on the selective effects of fishing, we predicted that largemouth bass inhabiting FPAs would have greater metabolic scope, as well as lower HPI axis-reactivity to an angling capture and air exposure stressor. To address the second objective, we quantified capture rates for largemouth bass inside versus outside each FPA using a common team of anglers. Based on a number of factors including angling experience, learning, social learning and potential differences in physiological properties, we predicted that rates of fish capture would be higher inside the FPA relative to angling sessions outside the FPA. This study is one of the first to quantify how spatial protection in the form of FPAs can provide evolutionary-enlightened benefits. The results of this study will help define the impacts that recreational angling may be having on the evolutionary trajectory of wild fish populations, and the potential role of individuals in protected areas to act as benchmarks for angling selection research.

## Methods

### Study site

All work was conducted on three inter-connected lakes within the Rideau lakes system (Ontario, Canada) including Opinicon (OP) lake, Newboro (NB) lake, and Big Rideau (BR) lake (Figure 1). Each lake has a self-sustaining largemouth bass population and all are well known by local and non-resident anglers for their high quality angling potential (Hogg *et al.*, 2010). Furthermore, BR lake supports the greatest angling pressure (measured as angling effort/day) of all lakes in eastern Ontario (Hogg *et al.*, 2010). Importantly, these lakes also house FPAs that were established in the 1930s and 1940s by the Ontario Ministry of Natural Resources and Forestry (OMNRF, formerly Lands and Forests; Ontario Department of Game and Fisheries, 1946; Figure 1). All forms of recreational angling are prohibited year-round within the borders of the FPA, whereas the remaining areas of the lake have been open-access to recreational angling and some small scale commercial harvest operations through time (Larocque *et al.*, 2012). Across lakes, the FPAs vary considerably in size relative to the total surface area of the lake. Specifically, the surface area of OP is 8.66 km^2^ with a cumulative FPA coverage of approximately 1.0 km^2^ (11.5% FPA coverage), the surface area of BR lake is 45.36 km^2^ with an FPA coverage of 0.57 km^2^ (1.2% FPA coverage), and the surface area of NB lake is 17.01 km^2^ with a cumulative FPA coverage of approximately 3.33 km^2^ (20% FPA coverage).

**Table 1:** Results of linear mixed effects models examining the factors influencing various physiological characteristics, as well as CPUE, of largemouth bass (*Micropterus salmoides*) residing within and outside FPAs, across three study lakes (OP, BR and NB lakes)

	Estimate	SEM	*df*	*t*	*P*	*R* ^2^_m_	*R* ^2^_c_
Baseline cortisol							
Intercept	3.42	0.62	2.24	5.48	0.024	<0.001	0.24
Location	−0.08	0.32	122.0	−0.25	0.80		
Maximum cortisol							
Intercept	70.90	11.358	2.56	6.24	***0.013***	<0.001	0.11
Location	2.97	8.46	122.0	−0.35	0.73		
Cortisol responsiveness							
Intercept	67.26	7.66	2.72	8.39	***0.005***	<0.001	0.08
Location	1.17	6.36	122.0	0.184	0.85		
Baseline glucose							
Intercept	2.71	0.07	3.26	38.703	***<0.001***	0.001	0.05
Location	−0.03	0.07	149.0	−0.44	0.66		
Maximum glucose							
Intercept	6.08	0.30	3.61	20.511	***<0.001***	<0.001	0.03
Location	−0.15	0.32	149.0	−0.46	0.64		
Glucose responsiveness							
Intercept	3.37	0.26	4.33	13.115	***<0.001***	<0.001	0.01
Location	−0.121	0.32	149.0	−0.38	0.71		
Resting metabolic rate							
Intercept	27.0	5.3	2.8	5.1	0.18	0.04	0.22
Location	−7.3	4.2	57.3	−1.7	0.09		
MMR							
Intercept	33.58	3.35	5.94	10.04	***<0.001***	0.02	0.04
Location	5.56	4.57	58.10	1.22	0.23		
AS							
Intercept	36.340	3.00	59.0	12.116	***<0.001***	0.073	0.07
Location	11.20	4.41	58.30	2.55	***0.0134***		
CPUE							
Intercept	8.23	2.66	2.27	3.10	0.08	0.259	0.689
Location	6.86	1.69	17.1	4.06	***<0.001***		

Bold, italicized, p values indicated statistical significance at α ≤ 0.05

Fixed effects for the models included ‘location’ (FPA and main-lake areas). Models included ‘Lake’ as a random effect. R^2^_m_ refers to r^2^ values for models without random effects, while R^2^_c_ refers to conditional r2 values, which include random effects in the model.

### Stress responsiveness experiment

Largemouth bass used in the stress responsiveness experiment were captured between July 20–27, 2015, by rod-and-reel angling (OMNRF permit no. 1082340) using a range of different soft-plastic lures typical of bass angling (i.e. worms, creature baits, and frogs) in an effort to maximize the variation in fish behaviour (Wilson *et al.*, 2015). Due to the logistical challenges associated with the habitats sampled, other forms of fish capture (e.g. trap netting and electrofishing) were not possible. Owing to the possibility of largemouth bass moving into/out of the FPA, sampling within the FPAs was conducted at the furthest possible point of access from FPA boundary lines, and angling outside the FPA was conducted far from the FPA (see Zolderdo *et al.*, 2019). Once hooked, fish were reeled in and hoisted from the water using a rubber-meshed landing net to reduce potential tissue damage, and placed in a foam-lined trough devoid of water. A blood sample (approximately 1 ml) was withdrawn from the caudal vasculature using a 21-gauge needle and a 3-ml vacutainer syringe containing lithium heparin (B.D. Vacutainer, Franklin Lakes, NJ) within the first 60 s of the fish being in the trough to provide baseline values for plasma glucose and cortisol. Largemouth bass were then subjected to 3 min of air exposure (a period of time that is sufficient to elevate plasma cortisol levels; Lawrence *et al.*, 2018). During this time, fish were measured (total length (TL) to the nearest mm) and weighed (to the nearest g), before being transferred to cylindrical bags with two permeable mesh endcaps that were submerged alongside the research boat for 27 min. Following the 27-min holding period (i.e. a period determined to achieve a maximal cortisol response in bass; McConnachie *et al.*, 2012), largemouth bass were removed from the recovery bags and a second blood sample was withdrawn using the procedure outlined above. This procedure allowed us to quantify the magnitude of the cortisol stress response for each individual (McConnachie *et al.*, 2012; Louison *et al.*, 2017). After collection of the second blood sample, fish were released. Fish used in this experiment did not differ in size (mm) across lakes, or between sample locations (Table 1A). Experimental protocols were approved by the Carleton University Animal Care Committee (AUP no. 104288) in compliance with the guidelines of the Canadian Council for Animal Care.

Blood samples were processed immediately aboard the research boat. Blood glucose levels for both the initial and post-stressor samples were measured using a handheld point-of-care blood glucose meter (Accucheck Compact Plus, Roche, Basel, Switzerland), a technique that has been validated for fish (Stoot *et al.*, 2014). The remaining blood was centrifuged for 2 min (2000*g*; Mandel Scientific, Guelph, ON, Canada) and plasma and red blood cells were transferred into separate microcentrifuge tubes and flash frozen in liquid nitrogen for future analysis of cortisol levels. Plasma cortisol concentrations were analysed using a commercial radioimmunoassay kit (MP Biomedicals, Orangeburg, NY) following the methodology of O’Connor *et al.* (2009). Inter- and intra-assay coefficients of variation for the cortisol RIA were 14.5% and 7.9%, respectively.

### Metabolic phenotype experiment

All fish used in the metabolic phenotype experiment were captured between 18 July and 31 August 2017, in the same manner and locations as fish used in the stress responsiveness experiment. For this study, however, all captured largemouth bass were transported in coolers (dissolved oxygen saturation never dropped below 70% during transport; Handy Polaris, OxyGuard, Farum, Denmark) by boat to the Queens’ University Biological Stations (QUBS). At QUBS, largemouth bass were held overnight (12–18 hrs) in 200 L flow-through tanks supplied with ambient OP water at a rate of approximately 18 L/min to recover from handling stressors and to enter a postabsorptive state prior to experimentation (Beamish, 1972; McConnachie *et al.*, 2012). Fish used in this experiment did not differ in size (g) between sample locations within a lake. However, fish were approximately 15% larger in BR lake, regardless of capture location, as compared to fish sampled from OP and NB lakes (see Appendix for details). Similarity, Fulton’s condition factor did not differ between sample locations within a lake, but fish from BR lake had higher condition factors on average than fish from OP and NB lakes (Table 1A).

All metabolic assessments were performed using static, intermittent-flow respirometry (Loligo Systems™, Tjele, Denmark) following methods outlined by Redpath *et al.* (2010), with a few modifications. Briefly, after the overnight acclimation period (approximately 7:00 pm to 8:00 am), each fish was removed from the holding tank and placed into a 100 L circular tank, where it was exercised to exhaustion via manual chasing and tail pinches (Louison *et al.*, 2017). Largemouth bass were deemed to be exhausted when they stopped responding to the stimulus. The fish was then removed from the exercise tank and held in a rubberized net for 1 min of air exposure, before being placed into one of four 11.78 L respirometry chambers submerged within one of two ~ 200 L tanks equipped with multiple air stones to ensure oxygen saturation remained at 100% throughout the entire testing period. The measurement cycle was set to 10 min ‘flush’ period, 3 min ‘wait’ period, and 10 ‘min’ measurement phase, which allowed r^2^ values for each data point to be > 0.9 (Svendsen *et al.*, 2016). It is important to note that each cycle produced one data point. Measurements of oxygen saturation in each chamber were taken every 5 s during the measurement phase by a fiber-optic probe. The rate of oxygen consumption (M_O2_, in mg O_2_ consumed kg^−1^ fish h^−1^) was calculated as the slope of the decline in oxygen concentration during each measurement period, and respirometry volume was corrected for fish volume prior to each trial.

The highest individual M_O2_ value obtained from the measurement cycles was taken as the fish's maximum metabolic rate (MMR) and was almost exclusively obtained within the first measurement cycle. Largemouth bass were left undisturbed within the respirometry chambers overnight to collect standard metabolic rate (SMR) data, calculated as the mean of the five lowest M_O2_ values (Nelson and Chabot, 2011; Louison *et al.*, 2017). This approach provides a representative estimate of SMR, as fish are allowed to fully acclimate and enter a resting state within the respirometry chambers prior to data collection (Clark *et al.*, 2013; Chabot *et al.*, 2016a, 2016b). Moreover, calculating SMR by averaging five of the lowest M_O2_ values provides an accurate depiction of the minimum obligatory oxygen requirements of a quiescent fish (Chabot *et al.*, 2016b).

Aerobic scope (AS) was calculated as the difference between MMR and SMR (Redpath *et al.*, 2010; Chabot *et al.*, 2016b; Louison *et al.*, 2017). Fish were promptly removed from the respirometry chambers the following morning (between 7:00 am and 8:00 am), and released, and a new set of measurements was initiated with four new fish. During respirometry work, largemouth bass were assigned to a respirometer chamber in a randomized fashion. Furthermore, fish from different lakes, as well as FPA and main-lake fish, were run concurrently when possible during each trial. All equipments (chambers, pumps and tubing) were sterilized regularly with a 10% bleach solution, and background respiration was evaluated periodically within each of the respirometry chambers and found to be negligible (Chabot *et al.*, 2016b). Each tank was equipped with water heaters to ensure water temperatures remained at 22°C to 25°C during the study period to match the ambient lake water temperatures within each of the replicate study lakes.

### CPUE

CPUE was calculated separately for FPA and main-lake areas based on the number of largemouth bass ≥200 mm caught per hour angling for all fish sampled during the stress responsiveness experiment. Approximately 55 h of angling time (17:46:30 within FPAs, and 36:50:16 within main-lake areas) was needed to capture the 193 largemouth bass used in this study (*n* = 98 for FPAs, *n* = 95 for main-lake areas; see Appendix for details). Because multiple areas were fished within each lake during data collection, CPUE data were calculated as individual blocks of time during which angling occurred; angling start time was noted once angling began, and stopped once the last fish was captured, for each area fished. Calculating CPUE in this manner enabled data to be standardized through the removal of biases including, but not limited to, travel time between areas and initial equipment set up time upon arrival in new fishing areas. However, the start time for 6 of the 21 fishing sessions (*n* = 1 FPA, *n* = 5 main-lake areas) was not recorded, and the time of first fish capture was recorded as the start time for those fishing sessions. Angling was conducted from a single boat using the same four anglers and the same fishing tactics for each angling session.

### Data analysis

Differences in physiological variables were quantified in two different ways using R version 3.6.3 (R Core Team 2020). First, physiological variables, as well as CPUE data, were compared for fish captured inside FPAs against those captured in main-lake areas using a linear mixed-model approach (Bolker *et al.*, 2009; Bolker 2015). We used a mixed modeling approach to compare fish from FPAs and main-lake areas because it allowed us to treat ‘lake’ as a random effect. This approach allowed us to consider the lakes we used as a random sample drawn from a larger ‘population’ of lakes (Bolker 2015), thereby allowing us to combine information across lakes and expand the scope of inference from our analyses beyond our three sites (Bolker *et al.*, 2009; Bolker 2015). Given that nested factors are typically conceptually random factors (Quinn and Keough, 2002), we chose not to nest our main effect (lake location) within our random lake variable so as to avoid violating model criteria (Schielzeth and Nakagawa, 2013). Mixed models were analysed using the ‘lme4’ package (version 1.1-21) (Bates *et al.* 2015), and the ‘lmerTest’ package (version 3.1-1) (Kuznetsova *et al.*, 2017). If a significant difference was detected in a mixed model, Tukey multiple-comparison tests were performed with estimated marginal means (least-squares means) using the ‘emmeans’ package (Version 1.4.4) (Searle *et al.*, 1980; Lenth, 2016). Marginal and conditional r^2^ values were generated using the ‘MuMIn’ package (version 1.43.15) (Barton, 2019).

In cases where grouping variables had fewer than five levels (as for our lake variable), uncertainty exists as to whether that variable should be treated as a fixed or random effect (Bolker *et al.*, 2009; Bolker 2015). To acknowledge this uncertainty, we supplemented our mixed models with a two-way analysis of variance (ANOVA) that did not use random effects. For these two-way ANOVAs, the main effects in the models were lake (OP, NB and BR), location (FPA vs main-lake area), and their interaction. If a significant fixed effect was detected in the ANOVA model, Tukey multiple-comparison tests were again performed using ‘emmeans’ (Searle *et al.*, 1980; Lenth, 2016).

All models were validated using standard techniques that included generating quantile-quantile plots to quantify normality, fitting residuals versus fitted values to verify homogeneity, and examining residuals versus each explanatory variable to check for independence (Zuur *et al.*, 2009). The presence of potential influential data points was also assessed (Zuur *et al.*, 2009). In the event that normality or variance assumptions were not met, data were rank transformed, models were re-run, and assumptions were confirmed (Conover and Iman 1981; Iman *et al.* 1984; Potvin and Roff, 1993). All data are presented as mean ± standard error (SE) where appropriate, and differences were considered significant where α was < 0.05.

## Results

### Stress responsiveness experiment

**Figure 2:**
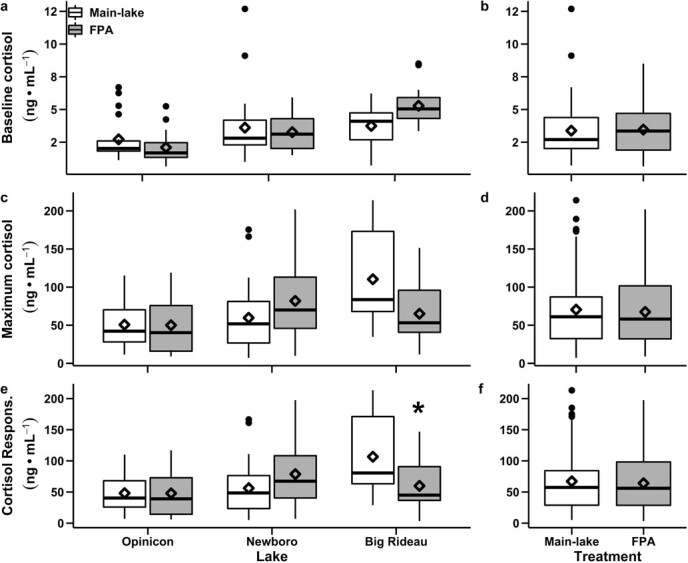
Comparison of stress response variables of largemouth bass (*Micropterus salmoides*) from within and outside freshwater protected areas (FPA) across study lakes. Panel (a) presents comparisons of baseline cortisol concentrations among study lakes, (c) presents comparisons of maximum cortisol concentrations among study lakes, and (e) presents comparisons of cortisol responsiveness (maximum-baseline) among study lakes. Panels (b), (d), and (f) present the corresponding comparisons for all lakes considered together. Asterisk in (e) represent a significant difference at a level of α ≤ 0.05 between FPA and main-lake area populations. See Results for details.

When all study lakes were considered together, there were no differences in baseline cortisol, maximum cortisol, or cortisol responsiveness between fish captured within FPAs and fish captured from main-lake areas; these results were consistent for mixed model analyses (Table 1), as well as two-way ANOVA models (Figure 2 a–f, Table 2). When examined within lakes, the cortisol response of largemouth bass captured from the FPA in BR lake was almost half that of fish captured from main-lake areas (Figure 2 e; Table 2). No differences were observed in the glucose variables evaluated between locations, or across lakes (Table 1, 2 and 3).

**Table 2:** Results of ANOVA models examining the factors influencing various physiological characteristics, and catch-per-unit-effort (CPUE), of largemouth bass (*Micropterus salmoides*) residing within and outside freshwater protected areas (FPA) across three study lakes, including Opinicon (OP), Big Rideau (BR) and Newboro NB lakes.

	DF	Sum Sq	Mean Sq	*F*	*P*
Resting metabolic rate					
Lake	2	311	1555	5.842	***0.005***
Location	1	798	797.7	2.996	0.089
Lake:Location	2	358	178.8	0.672	0.515
MMR					
Lake	2	3010	1505	0.912	0.408
Location	1	2791	2791	1.691	0.199
Lake:Location	2	4068	2043	1.238	0.298
AS					
Lake	2	79.8	39.90	13.266	***<0.001***
Location	1	6856	6856	5.877	***0.018***
Lake:Location	2	5262	2631	2.255	0.114
Baseline cortisol					
Lake	2	79.80	39.90	13.226	***<0.001***
Location	1	0.20	0.20	0.068	0.795
Lake:Location	2	25.20	12.580	4.171	***0.017***
Maximum cortisol					
Lake	2	25 183	12 591	6.017	***0.003***
Location	1	277	277	0.132	0.716
Lake:Location	2	23 720	11 860	5.668	***0.004***
Cortisol responsiveness					
Lake	2	22 432	11 216	5.342	***0.006***
Location	1	292	292	0.139	0.709
Lake:location	2	25 112	12 556	5.980	***0.003***
Baseline glucose					
Lake	2	1.240	0.620	3.869	***0.023***
Location	1	0.027	0.027	0.170	0.681
Lake:location	2	0.164	0.082	0.512	0.60
Maximum glucose					
Lake	2	19.0	9.504	2.398	0.094
Location	1	0.8	0.773	0.195	0.659
Lake/location	2	5.0	2.510	0.633	0.532
Glucose responsiveness					
Lake	2	13.0	6.479	1.649	0.196
Location	1	0.50	0.510	0.130	0.719
Lake:location	2	3.70	1.874	0.477	0.622

(Continued)

**Table 2:** Continued

	DF	Sum Sq	Mean Sq	*F*	*P*
CPUE					
Lake	2	328.8	164.39	11.47	***<0.001***
Location	1	216.4	216.39	15.01	***0.001***
Lake/location	2	9.9	4.93	0.34	0.71

Bold, italicized, p values indicated statistical significance at α ≤ 0.05

Fixed effects for the models included ‘lake’ and ‘location’, as well as the interaction between lake and location.

**Table 3:** Data for blood glucose between largemouth bass (*Micropterus salmoides*) populations residing inside freshwater protected areas (FPA) and within main-lake areas across Opinicon (OP), Big Rideau (BR), and Newboro (NB) lakes.

	FPA			Main Lake		
Physiological variable	OP	BR	NB	OP	BR	NB
Baseline glucose (mmol∙L^−1^)						
N (fish)	20	23	32	22	26	30
Mean	2.6	2.7	2.9	2.7	2.7	2.8
SE	0.08	0.08	0.08	0.08	0.07	0.08
Maximum glucose (mmol∙l^−1^)						
N (fish)	20	23	32	23	26	30
Mean	5.4	6.5	6.6	5.8	6.1	6.3
SE	0.41	0.44	0.28	0.42	0.46	0.38
Glucose responsiveness (mmol∙L^−1^)						
N (fish)	20	23	32	22	26	30
Mean	2.8	3.8	3.7	3.2	3.5	3.5
SE	0.40	0.45	0.29	0.41	0.45	0.4

See Results for details

### Metabolic phenotype experiment

**Figure 3:**
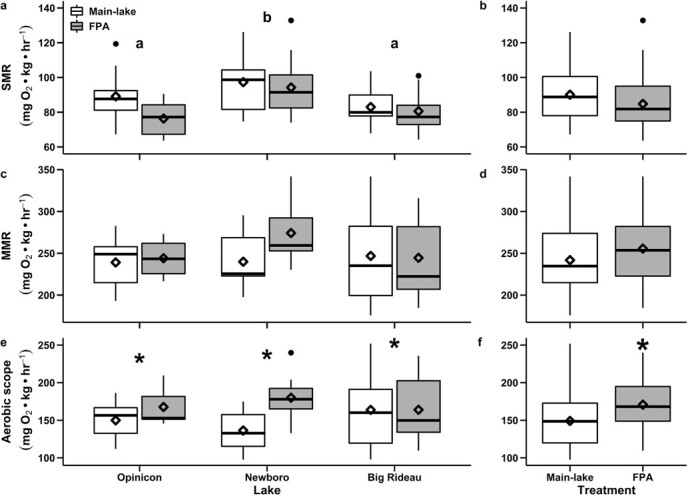
Comparison of metabolic variables of largemouth bass (*Micropterus salmoides*) from within and outside freshwater protected areas (FPA) across three study lakes. Panel (a) presents comparisons of standard metabolic rate (SMR) among study lakes, (c) presents comparisons of maximum metabolic rate (MMR), and (e) presents comparisons of aerobic scope (AS). Panels (b), (d), and (f) present the corresponding comparisons for all lakes considered together. Different lowercase letters in (a) denote a significant differences at a level of α ≤ 0.05 among treatment groups. Asterisks in (e) and (f) represent a significant difference at a level of α ≤ 0.05 between FPA and main-lake area populations.

When all study lakes were considered together, there were no differences in SMR between fish captured within FPAs relative to individuals captured from main-lake areas (Figure 3 a and b; Table 1). However, when examined within lakes, largemouth bass from NB lake showed SMR values that were approximately 16% greater than those from OP and BR lakes (Figure 3 a; Table 3). There were no differences in MMR across lakes or between FPA and main-lake regions (Figure 3 c and d; Tables 1 and 2). Fish residing inside FPAs showed AS values that were approximately 13% greater than those of fish captured in main-lake areas (Figures 3 e and f; Tables 1 and 2).

### CPUE

**Figure 4:**
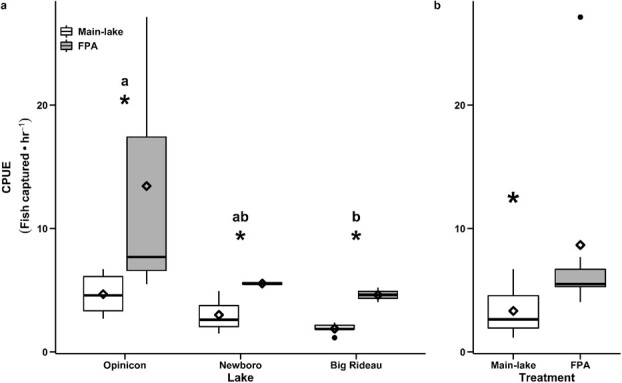
Catch-per-unit-effort (CPUE) data for largemouth bass (Micropterus salmoides) captured via angling techniques within (n = 98) and outside (n = 95) freshwater protected areas (FPAs) across each of the study lakes, including OP, BR and NB lakes. An asterisk represents differences in catch rates between FPA and main-lake areas at a given lake, while dissimilar letters show differences in catch rates across lakes.

Angler catch per hour of fishing effort (CPUE) was significantly higher inside FPAs relative to main-lake areas. Catch rates were approximately 1.5× higher inside FPAs of BR and OP, whereas catch rates were approximately 2.5× higher inside FPAs of NB Lake (Figure 4; Tables 1 and 2; also see Appendix for details).

## Discussion

Targeted selection pressures imparted through recreational fisheries can elicit genetic changes at a population level, resulting in the proliferation of suboptimal phenotypes (Philipp *et al.*, 2015; Duncan *et al.*, 2019; Koeck *et al.*, 2019). In the current study, largemouth bass captured from inside the freshwater protected areas (FPAs) had greater AS, which was largely driven by lower SMR values, compared to individuals captured from adjoining main-lake areas across three replicate lakes. This finding supports the hypothesis that angling may impair physiological phenotypes in wild fish populations (Hollins *et al.*, 2018; Duncan *et al.*, 2019). AS is defined as the difference between the maximum sustainable rate of aerobic metabolism and resting metabolic rate and sets the threshold for aerobic processes that can be performed simultaneously (Killen *et al.*, 2015; Treberg *et al.*, 2016; Hollins *et al.*, 2018). Reductions in AS at a population level suggest a reduced capacity to carry out essential life-history activities, which, in turn, may restrict key physiological functions, including the capacity to respond to dynamic environmental conditions (i.e. climate change) and result in fitness level implications (Duncan *et al.*, 2019). The lower AS of largemouth bass in main-lake areas may constrain their ability to perform energy-intensive activities (e.g. parental care; Cooke *et al.*, 2006; Sutter *et al.*, 2012) relative to individuals residing in the FPA on the same lake. Given that largemouth bass are sit-and-wait predators with relatively small home range sizes (Lewis and Flickinger, 1967; Demers *et al.*, 1996; Ahrenstorff *et al.*, 2009), individuals located deep within an FPA, such as those captured in the current study, may be naive to angling due to a reduced, or non-existent, exposure to angling. Indeed, a recent multi-year telemetry study has shown high occupancy rates (i.e. number of days inside the FPA boundaries) of largemouth bass within the BR FPA (Zolderdo, 2020). In that study, 50 largemouth bass were captured and tagged within the BRL FPA and monitored from 2016–2018 using acoustic telemetry. The study revealed that tagged largemouth bass exhibited inter-annual movements both inside and outside of the FPA, with the highest occupancy rates occurring during the early spring and summer seasons followed by a sharp decline in occupancy during the cold weather months (i.e. winter). Interestingly, the high occupancy rates during the spring–summer season happens to largely overlap with the legal fishing season for bass in the Rideau Waterway system (Zolderdo, 2020). In contrast, over the same time, largemouth bass from the main lake would have been exposed to angling, and associated harvest, incidental mortality (i.e. deep-hooking, angling stress; Siepker *et al.*, 2007), and/or angling-induced reproductive failure (Philipp *et al.*, 1997, 2023), which has the potential to remove certain phenotypes from the population. This marked difference in fishing pressure between fish inside and outside of the FPA is notable and thus it is unlikely that largemouth bass that we captured and studied in the FPA have experienced anywhere near the level of fishing mortality (including harvest) that those fish outside the sanctuary have experienced since the FPA was instituted over 70 years ago.

Although metabolic variables have not been found to drive angling vulnerability in largemouth bass (Louison *et al.*, 2017), exposure to angling-induced selection has consequences for the metabolic properties of largemouth bass. Individuals captured from lakes with no angling pressure (Hessenauer *et al.*, 2015) or individuals that were highly vulnerable to angling in an experimental setting (Redpath *et al.*, 2010) had higher metabolic performance relative to fish that had been subjected to angling, similar to the current study. The maintenance of high-performance AS phenotypes as a result of protection from human disturbance is consistent with both theory and empirical studies linking exploitation with functional trait diversity (Mouillot *et al.*, 2013; Diaz Pauli and Sih, 2017; Hollins *et al.*, 2018). For example, in red roman (*Chrysoblephus laticeps*), a commercially targeted marine fish, protection from hook-and-line fisheries in a marine protected area resulted in increased AS (Duncan *et al.*, 2019). Therefore, the high-performance AS phenotype observed in protected largemouth bass populations inhabiting FPAs may more closely represent the optimal threshold for aerobic performance indicative of natural selection processes in this species (Allendorf and Hard, 2009; Bull and Maron, 2016; Otto, 2018).

CPUE was significantly higher within FPAs relative to the adjacent main-lake areas across the three sites examined. For recreational fisheries, high CPUE is highly sought after by the angling community and resource managers alike. A number of conditions can interact to influence capture rates. For example, habitat and prey availability can interact to influence CPUE rates, because greater habitat availability/quality can support a higher density and diversity of food resources, which correlates inversely with capture likelihood in largemouth bass. Specifically, Keiling *et al.* (2020) observed higher largemouth bass capture rates in systems with lower prey resource availability. However, in the lakes examined in the present study, not only is habitat similar between FPA and main-lake areas, but prey resources are greater inside FPAs relative to the main-lake areas (Zolderdo *et al.*, 2019). Thus, the higher CPUE in FPA populations likely was not related to habitat, or differences in prey availability. Increased capture rates could reflect reduced experience with fishing lures resulting from a low, or non-existent, exposure to fishing lures relative the fish in the main-lake areas (Louison *et al.*, 2019). Higher population densities of largemouth bass inside FPAs also may have influenced CPUE rates (Zolderdo *et al.*, 2019). However, CPUE may not accurately reflect population density because capture rates can remain high even in systems with low population densities owing to habitat aggregation processes (Dassow *et al.*, 2020). It is also important to note that some angling trips were short owing to high catch rates thereby limiting the number of replicates of fishing excursions, and also that variance in CPUE was high within some sites. Despite these caveats, the difference in CPUE between FPAs and main-lake areas was pronounced. Regardless of the underlying mechanism, angler catch rates were almost twice as high within FPAs compared to those in main-lake areas despite standardizing the anglers engaged in fish capture.

No differences were detected in baseline cortisol, peak cortisol post-stressor, or cortisol responsiveness when all FPA and main-lake populations were considered together. Cortisol responsiveness previously was identified as the strongest driver of angling vulnerability in largemouth bass (relative to behavioral metrics such as boldness or exploration), with individuals exhibiting lower rises in plasma cortisol following a stressor being more likely to be captured via angling (Louison *et al.* 2107). Furthermore, HPI-axis sensitivity is heritable, and correlated to certain behavioural traits linked to angling vulnerability including bolder personality types (Øverli *et al.*, 2002; Oswald *et al.*, 2012; Wilson *et al.*, 2015; Lennox *et al.*, 2017; Koeck *et al.*, 2019). These bold behavioural types also were correlated to high AS phenotypes (Killen *et al.*, 2014; Binder *et al.*, 2016). Despite the lack of detectable differences when all lakes were combined, differences in cortisol values were observed for BR lake when it was examined individually, with individuals from the FPA having lower cortisol responsiveness than those from main-lake areas. This finding is consistent with current research and theory linking angling vulnerability with fast-POLS phenotypes. For instance, individuals with a fast-POLS profile express bolder behaviours coupled with reduced HPI-axis responsiveness (Réale *et al.*, 2010). This profile fits with the high-performance AS phenotypes observed within FPAs, as well as recent work showing increased parental aggression in FPA largemouth bass (Twardek *et al.*, 2017). It is unclear why this finding was isolated to BR lake, but it may reflect specific FPA traits. For example, the FPA on BR lake has the most restrictive entrance boundary of the FPAs examined (Zolderdo *et al.*, 2019), which may increase the protective capacity of the FPA, or alternatively, reduce fish migration in/out of the FPA, potentially isolating this FPA bass population to a greater extent than those of the other locations examined. However, because cortisol responsiveness is directly correlated to capture likelihood in largemouth bass, the establishment of FPAs may protect proactive stress coping phenotypes (lower cortisol responsiveness), which, in turn, may increase capture potential.

Although fisheries-induced selection remains the most parsimonious explanation for the observed trends in metabolic parameters and cortisol responsiveness, other factors may have played a role. For example, it is possible that environmental differences (e.g. water chemistry; Pickering and Pottinger, 1987) may have contributed to the observed physiological differences. However, all fish were collected from similar habitats within both FPA and main-lake areas to reduce any potential effects of environment. Also, previous research on the Rideau Waterway FPAs has noted a high degree of habitat similarity to adjacent main-lakes areas (Twardek *et al.*, 2017; Zolderdo *et al.*, 2019; Moynes *et al.*, 2020). Indeed, differences in AS were present across the three lakes despite this potential for inter-site variation. The use of angling as the sole method to collect fish may have caused sampling bias within the dataset. Angling can target specific personality types (Wilson *et al.*, 2015; Arlinghaus *et al.*, 2017; Cooke *et al.*, 2017), which has been linked to metabolic performance in certain species, including largemouth bass (Redpath *et al.*, 2010; Hollins *et al.*, 2018). For example, parental care capacity in male largemouth bass is positively correlated to aerobic metabolism, and also to angling vulnerability (Sutter *et al.*, 2012). However, all sampling for the current study was conducted outside the parental care period, when boldness and aggression are not drivers of angling vulnerability in largemouth bass (Louison *et al.*, 2017; Keiling *et al.*, 2020), and it is likely that female fish were captured in addition to males (although this was not quantified). Angling vulnerability outside the parental care period in largemouth bass is negatively correlated to food availability as well as prior angling experience (Hessenauer *et al.*, 2015; Louison *et al.*, 2019; Keiling *et al.*, 2020). As such, a sampling bias for naïve, hungry, individuals may have been present. Future work should use several sampling approaches (i.e. trap netting) to avoid possible sampling biases.

Freshwater habitats and the biodiversity they support are among the most imperiled ecosystems worldwide (Dudgeon *et al.*, 2006; Abell *et al.*, 2007; Reid *et al.*, 2019). The main threats facing freshwater ecosystems stem from anthropogenic resource uses (e.g. exploitive fisheries; Arthington *et al.*, 2016). Protected areas have become a cornerstone conservation strategy in terrestrial (Watson *et al.*, 2014; Chu *et al.*, 2018), and more recently, marine environments (Agardy, 1994; Halpern and Warner, 2002; Edgar *et al.*, 2014). Despite the host of benefits associated with the establishment of protected areas (Agardy, 1994; Halpern and Warner, 2002; Hilborn *et al.*, 2004), their application within freshwater systems has been limited, and consequently research into their effectiveness/utility is sparse (Suski and Cooke, 2007; Hermoso *et al.*, 2016; Acreman *et al.*, 2020). Previous studies have identified conservation benefits from the presence of FPAs, including improved biodiversity, increased species abundance, and greater reproductive output (Suski *et al.*, 2002; Hedges *et al.*, 2010; Zolderdo *et al.*, 2019).

The current study provides four additional benefits of freshwater FPAs. First, the current study demonstrates for the first time population level physiological benefits of FPAs through increased AS. Greater AS can increase the ability of an animal to perform work, ultimately reducing potential energy budgeting issues (e.g. growth and/or reproduction), which can result in fitness level benefits (Priede, 1985; Evans, 1990; Claireaux and Lefrançois, 2007). For example, reproductive fitness is positively correlated to AS in largemouth bass, where parental males with greater aerobic performance achieve higher reproductive success (Redpath *et al.*, 2010; Sutter *et al.*, 2012). Second, data from one site suggested that the presence of a FPA can reduce cortisol responsiveness, which may improve the ability of fish to deal with dynamic environmental conditions (e.g. climate change), because negative health consequences can arise from sustained activation of the HPI axis (Koolhaas *et al.*, 1999; Barton, 2002; Romero, 2012). Third, the presence of a FPA provides a physiological ‘baseline’ against which the impacts of angling, and other anthropogenic stressors, can be quantified. Fourth, spatial protection was found to significantly improve CPUE. Not only are high CPUE metrics sought after by resource managers and anglers alike, but are also potential markers of FIE (see Philipp *et al.*, 2015). However, for this fourth benefit to be realized, spillover of these angling-vulnerable fish outside of the FPAs would need to occur. These four benefits, when coupled with previous work on protected areas for largemouth bass and aquatic communities, should encourage managers and practitioners to consider implementing protected areas to enhance fisheries, particularly for species subjected to intensive recreational fishing. It is important to note that these benefits were achieved, not through closure of an entire lake system, but rather through the establishment of intra-lake exclusion zones, ranging from 0.5–18% of lake area. Of importance for future research is to identify specific habitat factors that contribute most significantly to protective capacity, to maximize conservation gains.

In conclusion, the current study provides evidence that FPAs provide benefits at a number of levels, including protecting high-performance AS phenotypes from angling selection and enhancing angler catch rates inside the FPA boundaries. The high degree of similarity in phenotypic traits observed in all three wild populations strongly supports the hypothesis that recreational angling may indeed alter functional trait diversity in wild fish populations. The findings presented here support the use of FPAs as a conservation strategy to counteract the selective potential of recreational fisheries practices. In addition, unexploited FPA populations can serve as benchmarks to further investigate the selective pressures imposed by recreational angling on wild fish. Furthermore, the current study highlights the effectiveness of exclusion zones to protect the more natural physiological state of exploited fish. Thus, lakes that house intra-lake FPAs may serve as holistic study systems to investigate other factors associated with human-use activities through comparative evaluations using unexploited FPA populations as a natural reference. Indeed, the results of the current study provide evidence linking metabolic performance with differing fisheries management strategies, likely as a result of fisheries-induced selection processes, which should encourage resource managers to consider protected areas as an evolutionary-enlightened management tool, especially for a fish species subjected to intensive recreational fisheries practices.

## Funding Statement

A.Z. was supported by the Ontario Graduate Scholarship program. This research was supported by Natural Sciences Engineerng Research Council (via a Discovery Grant, Steacie Award, and Strategic Project Grant to SJC) as well as the Canada Research Chairs Program. Additional support was provided by the BR lake Association and the Big Rideau Lake Environmental Fund.

## Declaration of Competing Interest

The authors have no conflicts to declare.

## Data Availability Statement

All data are accessible upon request from the corresponding author.

## Author Contribution Statement

**Aaron J. Zolderdo:** Conceptualization, Methodology, Data collection and analysis, Writing, Editing. **Alice E.I. Abrams**: Methodology, Data collection and analysis, Writing, Editing. **Michael J. Lawrence:** Methodology, Data collection and analysis, Writing, Editing. **Connor H. Reid:** Methodology, Data collection and analysis, Writing, Editing. **Cory D. Suski:** Conceptualization, Methodology Data collection and analysis, Writing, Editing. **Kathleen M. Gilmour:** Data collection and analysis, Writing, Editing. **Steven J. Cooke:** Conceptualization, Methodology, Data collection and analysis, Writing, Editing.

## A. Appendix

**Table 1A:** Data for sample size, weight (g), condition factor (Fulton's K), and CPUE for largemouth bass used in the different experiments in this study. Largemouth bass were captured from either inside FPA or from the main-lake areas across each of three study lakes: OP, BR, and NB

	FPA			Main-Lake		
Fish parameter data	OP	BR	NB	OP	BR	NB
Metabolic phenotype experiment						
*n* (fish)	7	13	12	9	11	9
Mean weight (g)	726	889	746	672	994	882
SE	60	69	61	43	103	72
Mean Fulton's K	195	231	203	186	252	225
SE	9.85	13.06	12.18	8.13	18.02	12.45
Stress responsiveness experiment						
*n* (fish)	19	17	26	19	17	28
Mean total length (mm)	354	337	348	356	361	336
SE	11	15	9	15	12	12
Glucose responsiveness experiment						
*n* (fish)	20	23	32	22	26	30
Mean total length (mm)	356	338	350	351	358	334
SE	11	11	7	14	9	11
CPUE						
*n* (fish)	33	32	33	33	31	31
Mean total length (mm)	348	351	350.01	347.62	363.26	330.63
SE	7	1	0.2	1	0.2	1
Mean fish caught per hour	13.45	4.63	5.55	4.69	1.89	3.00
